# CYLD: A Key Factor and Novel Predictive Biomarker of Cross-Resistance to Chemotherapy and Radiotherapy in Oral Squamous Cell Carcinoma

**DOI:** 10.7150/ijbs.138201

**Published:** 2026-10-02

**Authors:** Kou Yonemaru, Naoki Suenaga, Takeshi Masuda, Ryusho Kariya, Yuki Seki, Ryoji Yoshida, Hideki Nakayama, Yoshikazu Kuwahara, Fumihiro Nishimura, Kazutaka Oda, Jian-Dong Li, Hirofumi Jono

**Affiliations:** 1 Department of Clinical Pharmaceutical Sciences, Graduate School of Pharmaceutical Sciences, Kumamoto University, 2-2-1 Honjo, Chuo-ku, Kumamoto 860-8556, Japan.; 2 Institute for Advanced Biosciences, Keio University, 403-1 Nihonkoku, Daiboji, Tsuruoka-shi, Yamagata 997-0017, Japan.; 3 Division of Hematopoiesis, Joint Research Center for Human Retrovirus Infection, Kumamoto University, 2-2-1 Honjo, Chuo-ku, Kumamoto, 860-0811 Japan.; 4 Department of Oral & Maxillofacial Surgery, Faculty of Life Sciences, Kumamoto University, 1-1-1 Honjo, Chuo-Ku, Kumamoto, 860-8556 Japan.; 5 Division of Radiation Biology and Medicine, Faculty of Medicine, Tohoku Medical and Pharmaceutical University, 1-15-1, Fukumuro, Miyagino, Sendai, Miyagi, Japan.; 6 Department of Pharmacy, Kumamoto University Hospital, 1-1-1 Honjo, Chuo-ku, Kumamoto 860-8556, Japan.; 7 Center for Inflammation, Immunity and Infection, Institute for Biomedical Sciences, Georgia State University, Atlanta, GA 30303, USA.

**Keywords:** cylindromatosis (CYLD), cross-resistance, nuclear factor-kappa B (NF-κB), epidermal growth factor receptor (EGFR), targeted molecular therapy, oral squamous cell carcinoma (OSCC)

## Abstract

Cross-resistance to chemotherapy and radiotherapy is a major obstacle in cancer treatment and strongly associated with poor prognosis. Particularly, oral squamous cell carcinoma (OSCC) frequently exhibits such cross-resistance, limiting the efficacy of standard chemoradiotherapy. To unveil its molecular mechanism, we took advantage of a comprehensive proteomic analysis using clinically relevant radio-resistant OSCC cells, and identified distinct alterations in protein expression. Among the differentially expressed proteins, we focused on the down-regulation of cylindromatosis (CYLD), a deubiquitinating enzyme and tumor suppressor. Suppression of CYLD expression in OSCC cells led to accumulation of DNA damage and increased cross-resistance. CYLD down-regulation caused cross-resistance in OSCC cells, at least in part, via NF-κB hyperactivation. Notably, EGFR-TKI monotherapy demonstrated significant efficacy against cross-resistant OSCC* in vitro* and *in vivo*. Moreover, in patient-derived xenograft (PDX) models, CYLD expression might serve as a potential predictive biomarker for EGFR-TKI responsiveness in cross-resistant OSCC. These findings highlight the pivotal role of CYLD down-regulation driving OSCC progression via cross-resistance and offer new prospects for the clinical application of EGFR-TKIs, with CYLD expression serving as a potential predictive biomarker for OSCC patient with cross-resistance.

## Introduction

Cross-resistance to chemotherapy and radiotherapy, one of the most formidable challenges in cancer treatment, is significantly associated with poor prognosis 1. Particularly, oral squamous cell carcinoma (OSCC), which accounts for the majority of head and neck squamous cell carcinoma (HNSCC), is one of the most typical cancers exhibiting cross-resistance 2,3. The surgical resection for OSCC can impair oral function including deglutition, mastication, and speech, potentially leading to a significant decline in quality of life 4. Therefore, in addition to radiation treatment, non-invasive and effective chemotherapy is required as a standard therapy to maintain oral function. While chemoradiotherapies based on 5-Fluorouracil (5-FU) or cisplatin are commonly chosen and improve the survival rate of OSCC 5-7, recurrent or metastatic OSCC often exhibits the resistance to chemotherapy, which in turn leads to poor prognosis 5, 8-10. Moreover, emergence of radiation resistance is also a major cause of poor local control, recurrence, and poor prognosis 11-13. Although several studies have been conducted to identify factors involved in cross-resistance to chemotherapy and radiotherapy (such as drug efflux transporters, DNA repair and apoptosis pathways), breakthrough therapies based on these cross-resistance mechanisms remain largely unexplored.

In recent years, analysis of transcript variability such as RNA-seq has been widely employed to elucidate cancer molecular mechanisms. In fact, gene expression is subject to regulation at multiple levels, including post-transcriptional modifications, RNA interference, post-translational modifications, and protein degradation, such that mRNA expression doesn't necessarily correlate with protein expression. Proteome analysis involves the extraction and digestion of cellular proteins, followed by peptide measurement using liquid chromatography coupled with mass spectrometry (LC-MS/MS), allowing for the comprehensive identification and quantification profiling of proteins 14-15. Therefore, comprehensive understanding at the protein level through using proteome analysis is useful and essential for elucidating the molecular basis of cross-resistance. Taking full advantage of clinically relevant radio-resistant (CRR) cell lines, established by irradiation used in actual clinical practice 16, we attempted to elucidate clinically relevant cross-resistance mechanisms based on a comprehensive proteomic analysis to identify difference of proteins expression in CRR cells. The proteome analysis revealed the different proteins expression pattern of CRR cells comprehensively and identified several cross-resistance responsible proteins, including tumor suppressor cylindromatosis (CYLD).

CYLD, a deubiquitinating enzyme, is recognized as a tumor suppressor in many cancers 17-21. It was initially identified in familial cylindromatosis patients, and its mutation or loss of heterozygosity induced this benign human tumor 22-23. The CYLD protein contains ubiquitin-specific protease (USP) domain which cleaves lysine 63-specific polyubiquitin chains 24-25. Various association between CYLD and tumorigenesis have been reported, and several types of CYLD gene mutations are involved in tumorigenesis. More recently, our previous study demonstrated that the reduction of CYLD expression in OSCC patient tissues was significantly associated with the clinical features of deep invasion and poor overall survival 26.

In this study, based on our comprehensive proteomic analysis, we investigated the novel roles of CYLD in cross-resistance mechanisms of OSCC, and explored the possibility of developing a novel therapeutic strategy for patients with OSCC harboring cross-resistance to chemotherapy and radiotherapy.

## Material & Methods

### Cell lines and cell culture

Human oral squamous cell carcinoma cell line (SAS) was obtained from the Cell Resource Center for Biomedical Research, Cell Bank in Tohoku University, Sendai, Japan (cell ID TKG0470). The CRR cell line SAS-R was established from SAS as parental cells by irradiating X-ray with >60 Gy for 5 weeks at 2Gy per day, replicating the irradiation used in actual clinical practice 16. These cell lines were cultured in DMEM (Thermo Fisher Scientific, Waltham, MA, USA) containing 10% heat-inactivated fetal bovine serum (Thermo Fisher Scientific) in 5% CO_2_ at 37 ℃.

### Antibodies and reagents

Rabbit polyclonal anti-CYLD antibody (SAB4200060) was obtained from Sigma-Aldrich, Inc (Saint Louis, MO, USA). Rabbit monoclonal anti-gamma H2AX (phospho S139) antibody (ab81299) was obtained from Abcam (Cambridge, UK). Mouse monoclonal anti-β-actin antibody (A5441) was obtained from Sigma-Aldrich (St. Louis, MO, USA). Cisplatin (Nippon Kayaku, Tokyo, Japan), 5-FU (Sigma-Aldrich, Inc), Osimertinib (Selleck Chemicals, Houston, USA), Afatinib (Selleck Chemicals), NF-κB inhibitor, BAY 11-7085 (Abcam) and Bortezomib (Selleck Chemicals) were used in this study. For other reagents, commercially available special grades were used.

### Transfection with small interfering RNA

SAS cells were transfected with small interfering RNA (siRNA). At 24 h before siRNA transfection, the cells were seeded in 12-well plates (1.0 × 10^5^ cells/well), 24-well plates (0.5 × 10^5^ cells/well) and 35-mm dishes (2.0 × 10^5^ cells/well). The cells were grown and transfected with siRNA (10-50 nM) by using Lipofectamine 2000 (Thermo Fisher Scientific) according to the manufacturer's instructions. After transfection and incubation for 48 h, experiments were performed. The siRNA sequences targeting CYLD (siCYLD) were sense: 5'-GAUUGUUACUUCUAUCAAAtt-3' and antisense: 5'-UUUGAUAGAAGUAACAAUCtt-3' (Thermo Fisher Scientific). Silencer Negative Control siRNA (Thermo Fisher Scientific) was used as a control (siCon).

### Transfection with plasmids

At 24 h before plasmids transfection, the cells were seeded in 12-well plates (1.0 × 10^5^ cells/well), 24-well plates (0.5 × 10^5^ cells/well) and 35-mm dishes (2.0 × 10^5^ cells/well). The cells were grown and transfected with NF-κB-luciferase reporter plasmid (pGL4b vector; Promega, Madison, WI, USA) and CYLD plasmid by using Lipofectamine 2000 (Thermo Fisher Scientific) according to manufacturer's instructions. Empty vector (pcDNA3.0) was used as a control. After transfection and incubation for 48 h, experiments were performed.

### Irradiation

Irradiation was performed using a 150 KVp X-ray generator with total filtration though a 0.5 mm aluminum plus 0.1 mm copper filter (MBR-1520R; Hitachi, Tokyo, Japan). The dose rate (0.75Gy/min) was measured using a thimble ionization chamber (IC 17A; Far West Technology, Goleta, CA, USA).

### Cell viability assay

Cells were incubated in 24-well plates and treated with various anti-cancer agents (cisplatin: 2.5-10 μg/mL, 5-FU: 1.25-10 μg/mL, osimertinib: 5-20 μM, afatinib: 5-20 μM) in serum-free DMEM. After incubation for 48 h, 50 μL Cell Counting Kit-8 solution (DOJINDO LABORATORIES, Kumamoto, Japan) was added to each well of the plates. After incubation for 1.5 h, the plates were measured the absorbance at 450 nm by Emax SOFTmaxPRO (molecular devices, Tokyo, Japan).

### High-density survival assay

High-density survival (HDS) assays were performed as described by Kuwahara *et al*. 16. This approach is advantageous because, by allowing cells to be cultured for an extended duration post-irradiation, the HDS assay can thoroughly evaluate all cytotoxic responses. It encompasses not only early cell death mechanisms, such as apoptosis and necrosis, but also late cell death processes, including cellular senescence and mitotic death, which manifest several days later. Furthermore, while cell density may introduce bias in colony formation assays, the HDS assay employs high-density cell cultures, thereby mitigating issues such as delayed cell growth and is considered to more accurately reflect clinically relevant, in vivo-like phenotypes. An overview of the method is provided below. Cells (2.0 × 10^5^) were seeded in 35-mm dishes and incubated for 48 h. Cells were then exposed to 2, 5 or 10 Gy of irradiation. After 72 h of incubation, 10% of the cells were seeded in new 6-well plates and incubated for 72 h. Finally, the total cell numbers were counted via the Trypan blue dye exclusion assay.

### RNA isolation and real-time quantitative PCR

Total RNA extraction from cells was performed by phenol chloroform extraction method using TRIzol (Invitrogen, Waltham, Massachusetts, USA). Total RNA (200 ng) was reverse-transcribed to cDNA by the PrimeScript RT reagent (Takara Bio Inc, Shiga, Japan) according to the manufacturer's instructions. For quantification of mRNA, each PCR assay was performed using 1 μL of cDNA and each primer (10 μM) 0.3 μL, in a LightCycler System with SYBR Primer DimerEraser (Takara Bio Inc), with each reaction (with 10 μL samples) was performed under followed conditions: polymerase activation at 95 °C for 30 s followed by 40 cycles of 95 °C for 5 s, 55 °C for 30 s, 72 °C for 30 s. Human 18s ribosomal RNA was used as an internal control. Relative expression levels were determined by the ΔΔCt method. The sequences of each primer were as follows: Human CYLD, 5'-TCAGGCTTATGGAGCCAAGAA-3' (forward primer) and 5'- ACTTCCCTTCGGTACTTTAAGGA-3' (reverse primer); human 18s ribosomal RNA, 5'- CGGCTACCACATCCAAGGAA-3' (forward primer) and 5'-GCTGGAATTACCGCGGCT-3' (reverse primer); Human Fibronectin, 5'-CAGTGGGAGACCTCGAGAAG-3' (forward primer) and 5'-TCCCTCGGAACATCAGAAAC-3' (reverse primer).

### Protein extraction and Western blot analysis

Cells were washed once with ice-cold PBS and then lysed by adding RIPA Buffer (1×) with 1 mM PMSF (Cell Signaling Technology). Cell lysates were collected in tubes using cell scrapers (AGC TECHNO GLASS, Shizuoka, Japan) and centrifuged at 12,000 × g for 10 min after sonication. Supernatants were stored at -80 °C until use. Equal amounts of protein were fractionated via sodium dodecyl sulfate polyacrylamide gel electrophoresis and transferred to PVDF membranes (Millipore, Massachusetts, USA). Membranes were blocked with 2% non-fat dried milk (Cell Signaling Technology) in tris-buffered saline (0.05 M Tris, 0.138 M NaCl, 0.0027 M KCl, pH 7.8) containing 0.1% Tween-20 (TBS-T) and were then incubated overnight at 4 ℃ with primary antibodies in TBS-T containing 2% nonfat dry milk or 5% bovine serum albumin (Sigma-Aldrich). After membranes were washed with TBS-T, they were incubated for 1 h in horseradish peroxidase (HRP)-conjugated secondary antibodies at room temperature. After washing, specific protein bands were detected by using ECL Prime Western Blotting Detection Reagents (Amersham Life Science, Arlington Heights, IL, USA), according to the manufacturer's instructions.

### Immunohistochemical staining

Tissue samples for immunohistochemistry were fixed with 10% formalin before processing. Formalin-fixed specimens of clinical tissues were embedded in paraffin, cut into 5-μm-thick sections, and mounted on slides. These sections were dewaxed in xylene and then rehydrated in descending concentrations of alcohol. Endogenous peroxidase was blocked via a 30 min incubation of slides with 3% hydrogen peroxide. After slides were washed with PBS for 5 min, a non-specific staining blocking reagent (Nacalai tesque) was used for 10 min to block non-specific background staining, followed by overnight incubation at 4 °C with antibody against CYLD (1:50) diluted in PBS containing 1% BSA. Slides were rinsed with PBS for 5 min, incubated with secondary antibodies for 1 h, and washed again with PBS, and incubated with Liquid DAB Substrate Chromogen System (DAKO, Glostrup, Denmark) according to the manufacturer's protocol. Slides were lightly counterstained with hematoxylin for 30 sec before dehydration and mounting. Three persons took 10 photos per tissue in a blind state. CYLD protein expression levels in a total of 30 photos per tissue were quantified using ImageJ software.

### Proteome analysis by liquid chromatography-mass spectrometry (LC-MS/MS)

Whole cell lysate of SAS and SAS-R cells was prepared by phase transfer surfactant (PTS) method as described previously 27-28. Sodium deoxycholate (SDC), sodium N-lauroylsarcosinate (SLS), ammonium bicarbonate, dithiothreitol, iodoacetamide, mass spectrometry grade lysyl endoprotease, ethyl acetate, acetonitrile, acetic acid, methanol, trifluoroacetic acid (Wako, Osaka, Japan), modified trypsin (Promega, Madison, MA), and 4-(2-Aminoethyl) benzenesulfonyl fluoride hydrochloride (Nacalai, Kyoto, Japan) were used. Proteins were extracted with PTS solution (12 mM SDC, 12 mM SLS, and 100 mM TrisHCl (pH9.0) and ultrasonic crushed for 20 min. After incubating for 5 min at 95 °C, proteins in the supernatant solution were quantified by the BCA method using BCA Protein Assay Kit (Thermo Fisher Scientific). Proteins were reduced with 10 mM dithiothreitol for 30 min and alkylated with 50 mM iodoacetamide in the dark for 30 min at room temperature. The protein mixture was twofold diluted with 50 mM ammonium bicarbonate. And then, mixture was digested with Lys-C (1/50 sample weight) at room temperature for 3 h prior to the addition of trypsin (1/50 sample weight) and incubated at 37 °C for 20 h. An equal volume of ethyl acetate was added to the sample solution, and the mixture was acidified with 0.5% trifluoroacetic acid (final concentration). The mixture was shaken for 2 min and centrifuged at 15,600 × g for 2 min, and then the upper layer was removed by pipette, and dry up by a vacuum evaporator. The sample was suspended in 100 μL buffer A (5% acetonitrile, 0.1% TFA) and desalted with GL-Tip SDB (GL Science, Tokyo, Japan) 29-30. A TripleTOF 5600 (SCIEX, Framingham, MA, USA) equipped with a Dionex Ultimate 3000 RSLS (Thermo Fisher Scientific) was employed for nanoLC-MS/MS measurement. The injection volume was 5 μL, and the flow rate was 300 nL/min. A nano-trap column (100 μm ID, 2 cm length, packed with 5 μm Acclaim PepMap100C18, Thermo Fisher Scientific) and an analytical nanocolumn (75 μm ID, 25 cm length, packed with 2 µm Acclaim PepMap C18, Thermo Fisher Scientific) were used. The MS data was acquired with Analyst Software TF 1.7 (SCIEX). For peptide identification, data were acquired in the data-dependent acquisition mode and analyzed using ProteinPilot 4.5 (SCIEX) connected to the UniProt human reference proteome database (release 2017_11). For protein and peptide quantification, data were acquired in the data-independent acquisition mode (Sequential window acquisition of all theoretical fragment ion spectra: SWATH-MS) with a variable window for the precursor ions 31.

### Luciferase reporter assay

As above, cells were co-transfected with NF-κB-luciferase reporter plasmid and siRNA (or plasmid). After 48 h, samples were harvested with 1 × passive lysis buffer (PLB). Luciferase activities were determined with the Dual-Luciferase Reporter Assay System (Promega).

### Analyses of cell line-derived xenograft (CDX) model

BALB/c-nu/nu female mice (6 weeks old) were purchased from CLEA Japan and maintained at the Center for Animal Resources and Development of Kumamoto University. The animals were handled in accordance with the animal care policy of Kumamoto University. SAS and SAS-R cells were trypsinized, washed with serum-free medium, resuspended in phosphate buffered saline (PBS), and adjusted to a density of 1 × 10^7^ cells/100μl (50 μL PBS + 50 μL Matrigel). Then, the cell suspensions were subcutaneously injected into the backs of nude mice (n=4/cell line). When tumor volumes approached 100 to 150 mm^3^, the experiments were started (Day 0). The mice were treated with vehicle, 5-FU (20 mg/kg) every other day per intraperitoneal injection), osimertinib (5 mg/kg/day per oral administration) and afatinib (5mpk/day per oral administration). The tumor size was evaluated every 2 days by sequential caliper measurements of length (L) and width (W), and the tumor volume was calculated according to the formula (L × W2)/2. The Animal Care and Use Committee of Kumamoto University approved the protocols for all animal experiments.

### Analyses of patient-derived xenograft (PDX) model and PDX-derived cell lines

PDX-1 and PDX-2 derived from primary OSCC tumor and corresponding PDX-derived cell lines were prepared as described previously 32-33. This study has been approved by the ethical committee at Kumamoto University (approval No. 1427, No. 2389). The cells were grown in DMEM with 10% heat-inactivated fetal bovine serum in 5% CO2 at 37 °C.

### Statistical analysis

Student's t-test and analysis of variance were used to evaluate differences between the two groups. P-values of < 0.05 were considered statistically significant. Tukey-Kramer method was used to evaluate differences between more than three groups. All data are represented as the mean standard deviation (SD).

## Results

### SAS-R, a CRR cell line, acquires cross-resistance between radiation and cytotoxic drugs in OSCC

To confirm cross-resistance between radiation and cytotoxic drugs, we first performed the HDS assay to determine the cell survival rate of SAS and SAS-R cells by exposing to 2, 5 or 10 Gy of X-ray. The survival rate of SAS-R was higher than its parental cells, SAS, indicating SAS-R cells acquired the radiation resistance (Fig. 1A). In addition, we also assessed the sensitivity to anticancer drugs such as cisplatin (CDDP), 5-FU, and taxane-based drugs such as docetaxel (DTX) and paclitaxel (PTX), which frequently used for OSCC chemotherapy. As shown in Fig. 1B-E, SAS-R significantly exhibited the decreased cytotoxic sensitivities to all these anticancer drugs. Consistent with those *in vitro* results, the tumor sizes were markedly reduced in a CDX model transplanted with SAS cells by treatment of 5-FU, whereas it didn't reduce in CDX model transplanted with SAS-R cells (Fig. 1F-G). These results demonstrated that SAS-R acquired cross-resistance between radiation and anticancer drugs, indicating the clinical characteristics of recurrent and high-grade patients.

### Comprehensive proteomic analysis reveals tumor suppressor CYLD as a key factor for cross-resistance

To elucidate the factor causing the cross-resistance of OSCC, the proteome analysis targeting parental SAS and SAS-R cells was performed to reveal the differences in proteins expression pattern comprehensively. As shown in Fig. 2A, a total of 2,195 proteins exhibited increased proteins expression levels exceeding 1.5-fold in SAS-R cells compared to the parental SAS cells, whereas 3,477 proteins exhibited a decrease exceeding 1.5-fold. Furthermore, we extracted several proteins which match GO term 'regulation of intrinsic apoptotic signaling pathway' (GO:2001242) from them due to the process of apoptosis is regarded as the primary mechanism underlying radiation-induced cellular damage. A total of 52 proteins were identified and labeled (Fig. 2A), and these expression levels showed in heat map (Fig. 2B). Among them, we focused on CYLD, which was recognized as a tumor suppressor gene. CYLD regulates various intracellular signals such as NF-κB, Wnt/β-catenin, p38/mitogen-activated protein kinase, c-Jun N-terminal kinase, and Hippo and Notch signaling through deubiquitin activity 34-41. In recent years, reduction of CYLD expression is increasingly regarded as a critical factor contributing to malignant in various cancer including OSCC 17-21. Our previous study demonstrated that CYLD expression might be associated with malignant characteristics, such as, resistance & invasion, progression of T-classification/clinical Stage, and overall survival rate in OSCC 26. Indeed, CYLD expression at mRNA and protein levels in OSCC cell lines was reduced in SAS-R cells exhibited cross-resistance (Fig. 2C). The analysis focusing on HNSCC samples in Clinical Proteomic Tumor Analysis Consortium (CPTAC) and The Cancer Genome Atlas (TCGA), also confirmed the protein level of CYLD was decreased in grade3 patients compared with grade1 and 2 (Fig. 2D). Furthermore, the patients with low CYLD expression which underwent radiotherapy had shorter progression free interval (Fig. 2E). Thus, those results suggested that the acquisition of cross-resistance in OSCC might be caused by low CYLD expression.

### Low CYLD expression causes cross-resistance of OSCC

To clarify the roles of CYLD in cross-resistance, we first focused on the acquisition of radio-resistance in OSCC. SAS cells with CYLD knocked down by CYLD-specific siRNA (Fig. 3A and Supplementary Fig. 1A), exhibited significantly lower radiation sensitivities compared with control group (siCon) (Fig. 3B). Besides, siCYLD group also exhibited the decreased anti-cancer drug sensitivities such as 5-FU and cisplatin compared with siCon group (Supplementary Fig. 2A-B). Moreover, we also assessed the expression of γ-H2AX, an indicator for DNA double-strand break (DSB) in radiotherapy. In siCYLD group, the γ-H2AX expression after irradiation disappeared earlier than siCon group (Fig. 3C). In addition, γ-H2AX expression measured under same conditions in CRR cells, and its expression similarly disappeared earlier than parental cells (Fig. 3D), suggesting that low CYLD expression might activate DNA repair mechanisms, like homologous recombination repair (HRR). To further confirm the roles of CYLD, we generated CYLD overexpression (CYLD-OE) OSCC cells (Fig. 3E and Supplementary Fig. 1B). Interestingly, the CYLD-OE group exhibited increased sensitivities to radiation compared with group which was transfected with control group (pc-DNA) in SAS-R cells (Fig. 3F). Moreover, in CYLD-OE group, γ-H2AX expression remained longer than pc-DNA group in CRR cells (Fig. 3G), indicating that CYLD affected the activation of DNA repair mechanisms. Therefore, the low CYLD expression in OSCC cells could lead to activate the DNA repair mechanisms which suppressed DSB from radiotherapy and chemotherapy, resulting in cross-resistance.

### Molecular mechanisms underlying the cross-resistance caused by low CYLD expression

Previous studies have shown that CYLD negatively regulates NF-κB signaling pathway and has been closely related to the progression of the malignancy in several cancers 34-36. Additionally, NF-κB has been identified as a key factor in the development of drug resistance in OSCC 42. We thus sought to investigate the relationship between the NF-κB signaling pathway and radio-resistance in OSCC. As shown in Fig. 4A, NF-κB activity was significantly enhanced in siCYLD group compared with siCon group, indicating the low CYLD expression indeed induced NF-κB hyperactivation. Furthermore, pre-treatment of BAY 11-7085, an irreversible NF-κB inhibitor, remarkably increased the radiation sensitivities in siCYLD group (Fig. 4B), suggesting low CYLD expression might cause radio-resistance through NF-κB signaling pathway. Consistently, CRR cells independently exhibited lower CYLD expression and higher NF-κB activity than parental cells, and their radiation sensitivities were significantly increased when being pre-treated with BAY 11-7085 (Fig. 4C, D). Since CRR cells were established through long-term exposure to radiation, these cells are considered to more closely reflect radio-resistant cancer cells found in clinical patients; nevertheless, the association between NF-κB activation and radio-resistance was also observed in these cells. Given that CRR cells also showed low CYLD expression and hyperactivation of NF-κB, the reduction of CYLD might cause cross-resistance through NF-κB signaling pathway.

### EGFR-tyrosine kinase inhibitors are significantly effective for OSCC with cross-resistance

Although our results revealed the molecular mechanisms underlying the cross-resistance of OSCC at least in part, novel therapeutic strategies that are independently effective to CRR cells were still needed. We focused on the hyperactivation of several intracellular signaling pathways such as epidermal growth factor receptor (EGFR), PI3K/AKT/mTOR and NF-κB pathway induced by reduction of CYLD which may contribute to the malignancy in OSCC and thus evaluated the effects of their inhibitors on CRR cells 34-36, 43-46. In this study, no efficacy was observed for everolimus, a PI3K/AKT/mTOR inhibitor, and BAY 11-7085, an NF-κB inhibitor, on CRR cells (Fig. 5A-C). These findings suggest that NF-κB inhibitor has potential efficacy in combination with radiation therapy, as demonstrated in Fig. 4, but it is unlikely to exert therapeutic effects as mono-therapy. Intriguingly, as shown in Fig. 5A, D-E, EGFR-tyrosine kinase inhibitors (TKIs), such as osimertinib and afatinib showed remarkable effects on CRR cells, in contrast to the clearly limited effects of standard chemo-radiotherapy. It should be noted that EGFR-targeted molecular therapies such as gefitinib have been effective for CYLD-negative and poor prognosis OSCC 44. We further investigated the sensitivity of osimertinib and afatinib *in vivo*, by using CDX model established by subcutaneous injection of SAS-R cells and treated with these drugs orally. Consistent with the *in vitro* results, tumor volume of SAS-R cells was significantly reduced in CDX model treated with both osimertinib and afatinib (Fig. 5F). These results demonstrated that the EGFR-TKIs might be effective for recurrent cases with cross-resistance like CRR cells.

### Efficacy of EGFR-TKIs for patient-derived xenograft (PDX) models with cross-resistance by low CYLD expression

PDX models, generated by direct transplantation of patient tumor samples into immunocompromised mice, are recognized as the most clinically relevant *in vivo* cancer models 47. Finally, we generated PDX models derived from OSCC patient tumors (strain PDX-1 and PDX-2), and established PDX-derived primary cultured cells as the most relevant CRR cells. As shown in Fig. 6A-D, PDX-2 showed the resistance to the standard therapies, such as, radiation, CDDP, 5-FU, and DTX compared with PDX-1, exhibiting the cross-resistance between radiotherapy and chemotherapy. Interestingly, consistent with our results shown in Fig. 5, PDX-2 showed significantly higher sensitivity for EGFR-TKIs, such as, osimertinib and afatinib (Fig. 6E, F). To further support clinical results shown in Fig. 2D-E, PDX-2 with cross-resistance indeed exhibited lower CYLD expression than PDX-1 (Fig. 6G), suggesting the close association between low CYLD expression and cross-resistance. As expected, PDX-1 down-regulating CYLD expression acquired the cross-resistance (Fig. 6H, I), while PDX-2 overexpressing CYLD expression significantly increased the sensitivity of those treatments (Fig. 6J-K). Furthermore, to determine whether CYLD expression might serve as a predictive biomarker for EGFR-TKIs treatment, we assessed the CYLD expression of OSCC patient tumor tissues in PDX model by immunochemical staining. As a promising result, the OSCC patient tumor of PDX-2 showing both cross-resistance and high sensitivity for EGFR-TKIs, indeed exhibited low CYLD expression (Fig. 6L). Taken together, our results strongly supported that low CYLD expression might serve as a predictive biomarker of EGFR-TKIs treatment for cases with cross-resistance.

## Discussion

In patients with OSCC, chemoradiotherapy is an essential treatment option to protect patient's quality of life and extend healthy life expectancy by maintaining oral function. However, chemoradiotherapy often failed because of resistance, especially in recurrent and metastatic cases 5, 8-10. Hence, improving the sensitivity of chemoradiotherapy or developing novel therapeutic targets has become a critical clinical challenge in this field.

In this study, we sought to elucidate the mechanism of OSCC cross-resistance by conducting comprehensive proteomics analysis to examine protein expression changes in CRR cells (Fig. 2). In comparison with transcriptome analysis, proteomics analysis provides a more functionally relevant analysis of gene expression levels *in vivo*. Moreover, we were able to explore mechanisms of resistance more generally in term of using CRR cells. CRR cells were established by Kuwahara *et al*. through irradiating with >60 Gy for 5 weeks at 2Gy per day 16. This method replicated the irradiation used in actual clinical practice, and would be thus considered a close mimic of the actual radio-resistant cancer cells in patient's tumor. Interestingly, the CRR cells generated in this way also developed cross-resistance to anti-cancer drugs commonly employed in OSCC therapy (Fig. 1). Kuwahara *et al*. who first established CRR cells reported that CRR cells exhibited cross-resistance only to docetaxel and not to 5-FU and CDDP 48. We speculate that this discrepancy may be attributable to differences in the concentrations of anti-cancer drug exposure or in the methods used to assess cell viability. Although SAS-R cells were employed for the evaluation of cross-resistance in this study, further studies should confirm the validity of these findings in other CRR cell lines. As noted above, we investigated the factors underlying cross-resistance using a novel approach unlike those previously reported in this study. To select the most crucial factor for inducing cross-resistance from the numerous proteins exhibiting expression changes (Fig. 2), we focused on key intracellular signaling pathways which have been previously reported to be involved in resistance. Among those pathways, the EGFR pathway, which is overexpressed in 80-90% of HNSCC patients 49, appears to be of significance. OSCC patients with high EGFR expression exhibit increased rates of local recurrence after chemoradiotherapy 50-51. Previous studies have suggested that EGFR may activate the repair of radiation-induced DSB by promoting the formation of EGFR-DNA-PK complexes and enhancing ATM-mediated DNA-PK phosphorylation 52-53. Additionally, the PI3K/AKT/mTOR pathway, which is overexpressed in 37% of HNSCC patients 54-55, has been reported to be activated by NOX1 stimulation, leading to drug resistance 56. Furthermore, Wnt/β-catenin pathway is a common intracellular signaling pathway that regulates the proliferation in OSCC, and has been reported to induce resistance to cisplatin and 5-FU, as well as radiation resistance associated with the acquisition of cancer stem cell-like properties 57-59. Interestingly, NF-κB pathway is strongly associated with drug and radiation resistance when stimulated by factors such as HOXA1, AIM2 and GBP5 60-62. Therefore, multiple intracellular signaling pathways are involved in cross-resistance in OSCC. Importantly, CYLD is known to regulate each of the aforementioned pathways in addition to regulating the apoptosis response 34-37, 43-44, 63. Therefore, we focused on CYLD downregulation in CRR cells (Fig. 2).

CYLD, a deubiquitinating enzyme, is recognized as a tumor suppressor gene in several cancer types 17-21. Previous studies have reported that CYLD targets tumor necrosis factor receptor-associated factor (TRAF)2, TRAF6 and nuclear factor-κB essential modulator (NEMO), negatively regulate nuclear factor-κB (NF-κB) signaling pathway 34-36. In addition to NF-κB, other intracellular signals such as Wnt/β-catenin, c-Jun N-terminal kinase (JNK), p38/mitogen-activated protein kinase, and Hippo and Notch are also regulated by CYLD in immune and cancer cells 37-41. Various associations between CYLD and tumorigenesis have been reported, and several types of CYLD gene mutations are involved in tumorigenesis. More recently, CYLD dysfunction due to the reduction of its expression is strongly correlated related to tumorigenesis and poor prognosis. Massoumi *et al*. have suggested that the reduction of CYLD enhances tumor growth and invasion, and the overall survival is inversely correlated with CYLD expression in melanoma 17. Moreover, in many other cancers such as non-small cell lung cancer, hepatocellular carcinoma, basal cell carcinoma, breast cancer, glioblastoma and cholesteatoma, various studies have revealed a correlation between the reduction of CYLD expression and poor prognosis, including increased tumor growth, drug-resistance and invasive capacity 18-21. In particular, our previous studies demonstrated that the reduction of CYLD expression in OSCC patient tissues was significantly associated with the clinical features of deep invasion and poor overall survival 26. In addition, CYLD knockdown caused cisplatin resistance in OSCC cells due to suppression of apoptosis and intracellular accumulation of cisplatin by NF-κB hyper-activation 64. From the above, these reports suggest that the reduction of CYLD expression is a key factor in poor prognosis in OSCC. In this study, repeated irradiation resulted in the downregulation of CYLD expression in CRR cells. Our preliminary findings indicate that inhibition of DNA methylation partially restores CYLD expression, suggesting that epigenetic regulation mediated by DNA methylation may contribute to the CYLD downregulation following repeated irradiation. We will keep investigating the mechanism underlying the CYLD downregulation in the future. This study revealed that impact of CYLD downregulation extends beyond multiple anticancer drugs and radiation (Fig. 3, Supplementary Fig. 2). Thus, the reduction of CYLD is assumed to have a direct impact on the overall therapeutic response to chemoradiotherapy, which is a serious factor leading to a poor prognosis in OSCC.

We hypothesized that the NF-κB pathway is relevant as the underlying mechanism for resistance in OSCC (Fig. 4). NF-κB pathway is known as a key role in inflammation, immune response and oncogenesis. As described above, CYLD negatively regulates the NF-κB pathway by removing K63-linked polyubiquitin chains from key mediators of NF-κB activation, including TRAF2, TRAF6 and NEMO. Therefore, reduction of CYLD expression is expected to impair its deubiquitinating activity toward these substrates, leading to sustained activation of the NF-κB pathway. Since NF-κB activation is also known to exhibit anti-apoptotic effects in the cancer cells, NF-κB hyperactivation caused by loss of deubiquitinase activity of CYLD may contribute to the development of cross-resistance to chemotherapy and radiotherapy in OSCC. It is still unclear how CYLD downregulation-induced NF-κB hyperactivation triggers cross-resistance. However, it is widely known that NF-κB pathway suppresses apoptosis by inducing transcription of anti-apoptotic genes such as Bcl-2, Bcl-xL and XIAP 65-67. Moreover NF-κB pathway has also been reported to promote DNA repair pathway activation through upregulating BRCA1 and RAD51 68-69. It is logical to postulate that these effects may act synergistically to induce cross-resistance. Based on our findings, a novel therapeutic strategy combining NF-κB inhibitors with chemoradiotherapy might be advantageous. However, it should be noted that NF-κB inhibitor monotherapy failed to exert a therapeutic effect, and potential side effects remain a significant concern. Thus, we paid more attention to the efficacy of EGFR-TKIs as an effective monotherapy for cross-resistant OSCC (Fig. 5, 6). EGFR-TKIs demonstrated efficacy as monotherapy, resulting in fewer concerns about side effects still compared to the combination of NF-κB inhibitors and chemoradiotherapy mentioned above. However, although CYLD was downregulated in CRR cells, increasing phosphorylation of EGFR-associated proteins remains to be further confirmed (data not shown). In addition, we were unable to demonstrate the effectiveness of gefitinib, which had been effective in previous report in CYLD-specific Knockdown cells. The molecular mechanism for this difference is unclear, but it is probably attributed to a complex interaction of secondary changes other than CYLD, since CRR cells weren't CYLD-specific knockdown cells. This difference will certainly need to be further investigated in more detail for in the future. Taken together, because CRR cells are clinically relevant resistant cells, we postulate that osimertinib and afatinib should be more effective than gefitinib when actually being used in cross-resistant patients. Furthermore, the results in Fig.6 using the PDX models strongly supported that low CYLD expression might serve as a predictive marker for EGFR-TKIs efficacy. Therefore, EGFR-TKIs might be more effective for patients with resistant state induced by CYLD downregulation.

In summary, we demonstrated that the reduction of CYLD expression caused cross-resistance in OSCC. In addition, EGFR-TKIs were effective for cross-resistant OSCC cells based on the reduction level of CYLD expression. Taken together, in patients with low CYLD expression, conventional chemoradiotherapy may fail to achieve sufficient therapeutic effects, whereas EGFR-TKIs may represent a potentially effective therapeutic option. These findings suggest that CYLD may serve not only as a prognostic marker but also as a predictive marker for treatment response. In the future, assessment of the CYLD expression in tumor tissues and treatment selection based on its rate may warrant a novel strategy of personalized pharmacotherapy for recurrent and aggressive cases of patients with OSCC. CYLD-based therapeutic stratification may expand the limited treatment options currently available for patients with cross-resistant tumors.

## Supplementary Material

Supplementary figures.

## Figures and Tables

**Figure 1:**
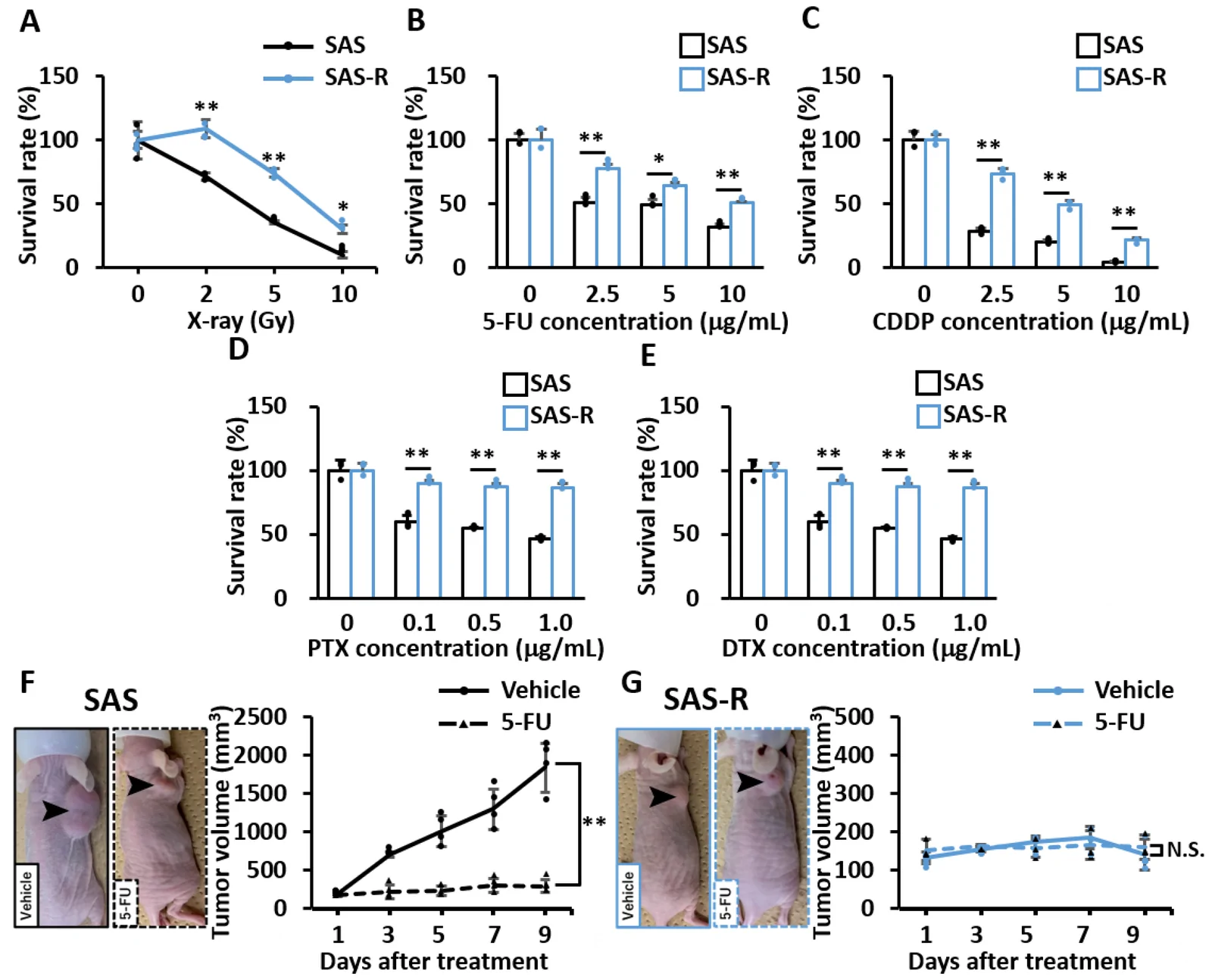
** SAS-R, a CRR cell line, acquire cross-resistance between radiation and cytotoxic drugs in OSCC.** (A) The cell survival rates were assessed after irradiation (0-10 Gy) by using HDS assay. (B-E) The cell survival rates were assessed after treatment of 5-FU (0-10 μg/mL), CDDP (0-10 μg/mL), DTX (0-1μg/ml), and PTX (0-1µg/mL) by using Cell Counting kit-8. (F-G) Image of mice at day 9 after reagents treatment. The tumor growth curves were measured after 5-FU treatment. Values are means ± S.D. of triplicate samples. *p<0.05, **p < 0.01 vs SAS group in Tukey-Kramer method. N.S.: not significant. Data are representative of 2-3 independent experiments.

**Figure 2:**
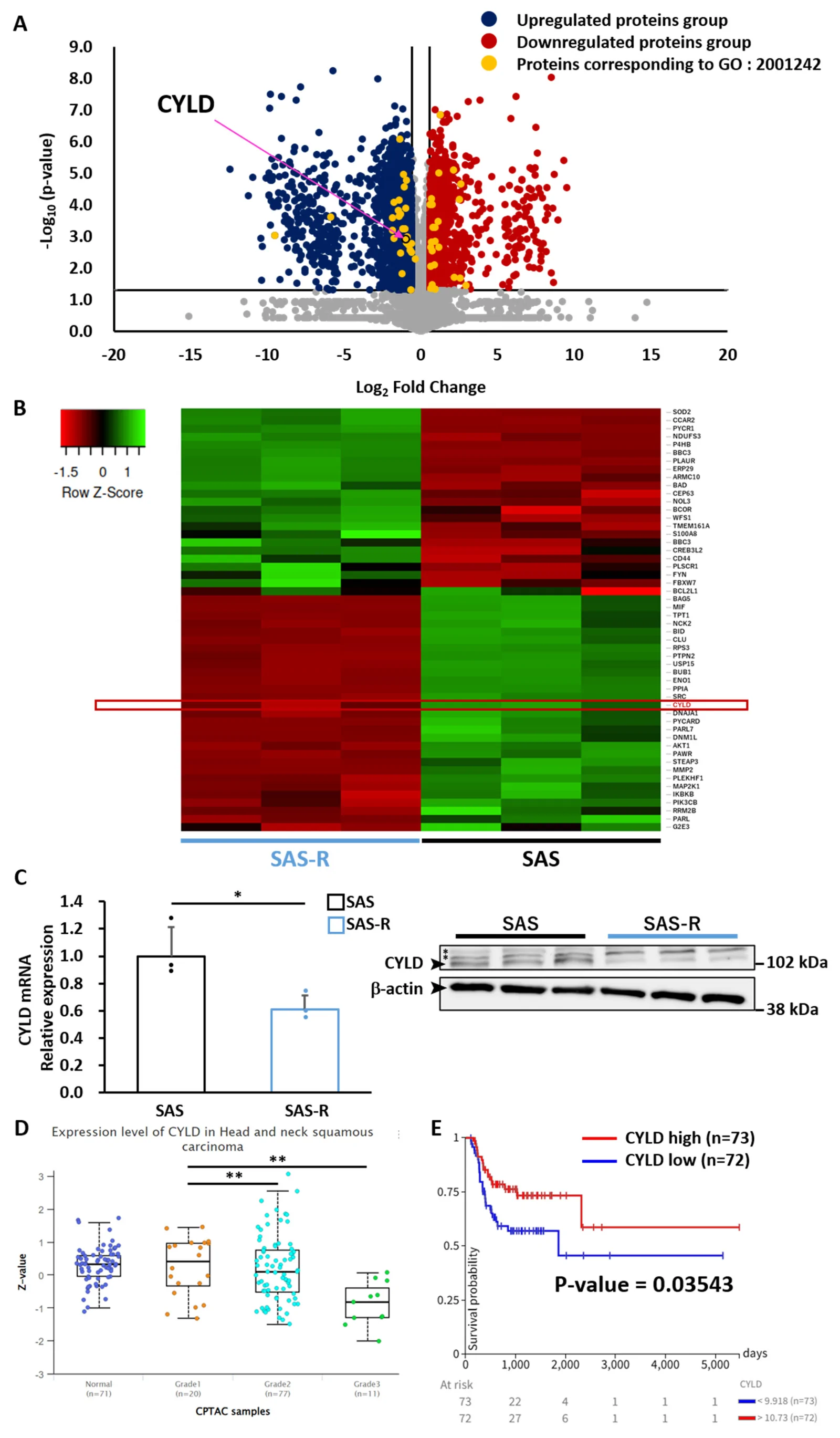
** Comprehensive proteomic analysis reveals tumor suppressor CYLD as a key factor for cross-resistance.** (A) Comprehensive changes in protein expression between SAS and SAS-R were assessed by proteome analysis. The arrow indicates CYLD protein. Volcano plot showing up- and down-regulated proteins in SAS-R compared with SAS. (B) Heat map shows expression of proteins corresponding to GO: 2001242. (C) Relative CYLD mRNA expression and protein were assessed by RT-qPCR and Western blot analysis. The asterisk (*) indicates a non-specific band. Values are means ± S.D. of triplicate samples. *p<0.05, **p < 0.01 vs SAS in Student's-t-test. Data are representative of 3 independent experiments. (D) Analysis of CYLD protein expression in normal and tumor tissue separated by grade from the CPTAC HNSCC patients. Values are means ± S.D. of triplicate samples. **p < 0.01 vs Grade 1 in Tukey-Kramer method. (E) Analysis of progression free interval in the TCGA HNSCC patients were underwent radiotherapy when divided into top 25% (CYLD high) and bottom 25% (CYLD low) of CYLD mRNA expression. P-value was calculated by log-rank test.

**Figure 3:**
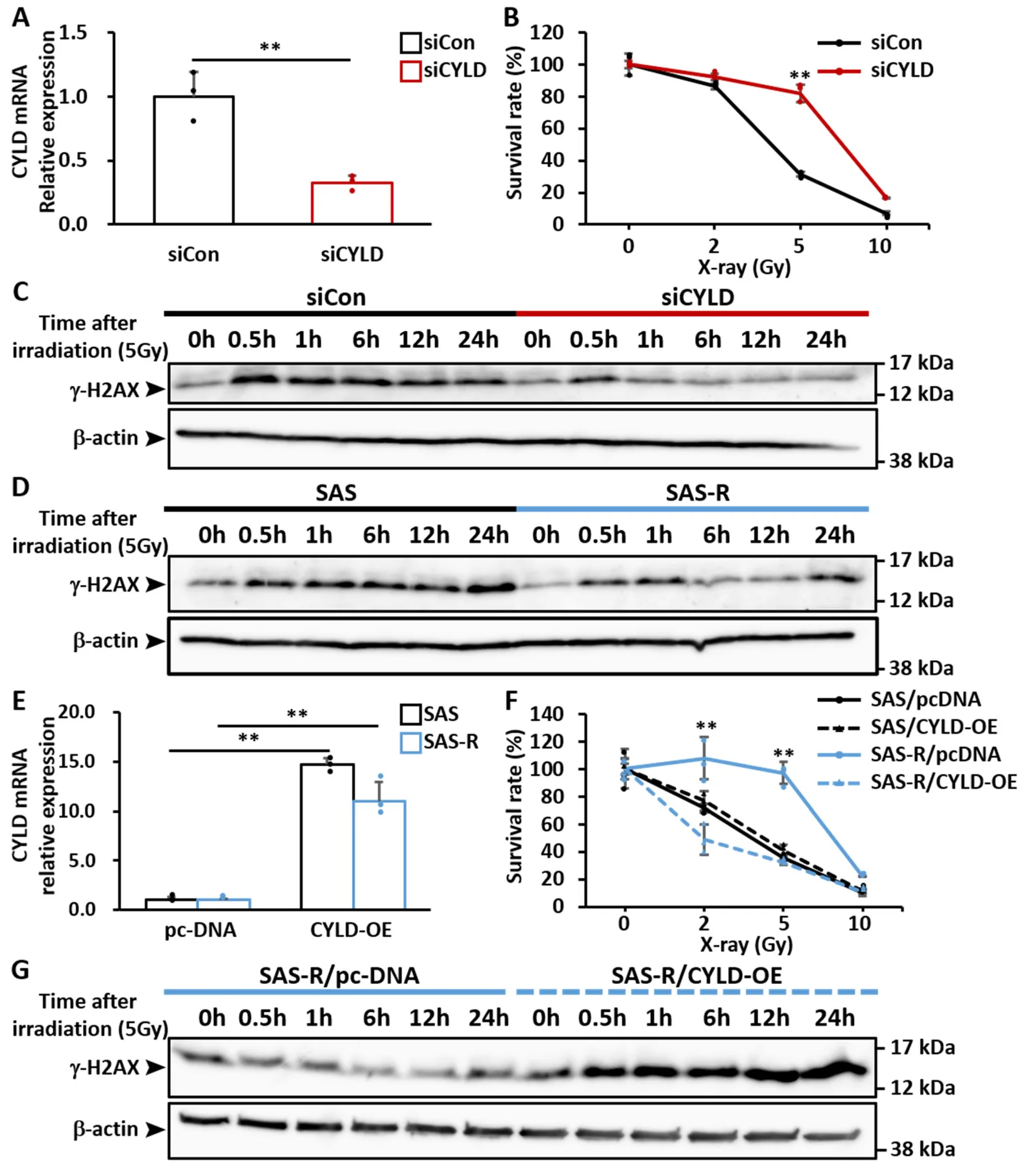
** Low CYLD expression causes cross-resistance of OSCC.** (A) SAS cells were transfected with control siRNA (siCon) or CYLD-specific siRNA (siCYLD). Relative CYLD mRNA expression 48h after siRNAs transfection were assessed by RT-qPCR. (B) The survival rates of siCYLD were evaluated after irradiation by using HDS assay. Values are means ± S.D. of triplicate samples. *p<0.05, **p < 0.01 vs siCon in Tukey-Kramer method. (C) siCYLD cells were irradiated at 5 Gy and cultured for 0.5, 1, 6, 12 and 24 h. Western blotting showing the levels of γ-H2AX. (D) SAS-R cells were irradiated at 5 Gy and cultured for 0.5, 1, 6, 12 and 24 h. Western blotting showing the levels of γ-H2AX. (E) SAS and SAS-R cells were transfected with control plasmid (pc-DNA) or CYLD (CYLD OE). Relative CYLD mRNA expression 48h after transfection with plasmids assessed by RT-qPCR. (F) The survival rates of CYLD-OE were evaluated after irradiation by using HDS assay compared with each cell transfected with pc-DNA. Values are means ± S.D. of triplicate samples. **p < 0.01 vs pc-DNA groups in Tukey-Kramer method. (G) CYLD-OE SAS-R cells were irradiated at 5 Gy and cultured for 0.5, 1, 6, 12 and 24 h. Western blotting showing the levels of γ-H2AX. Data are representative of 2-3 independent experiments.

**Figure 4:**
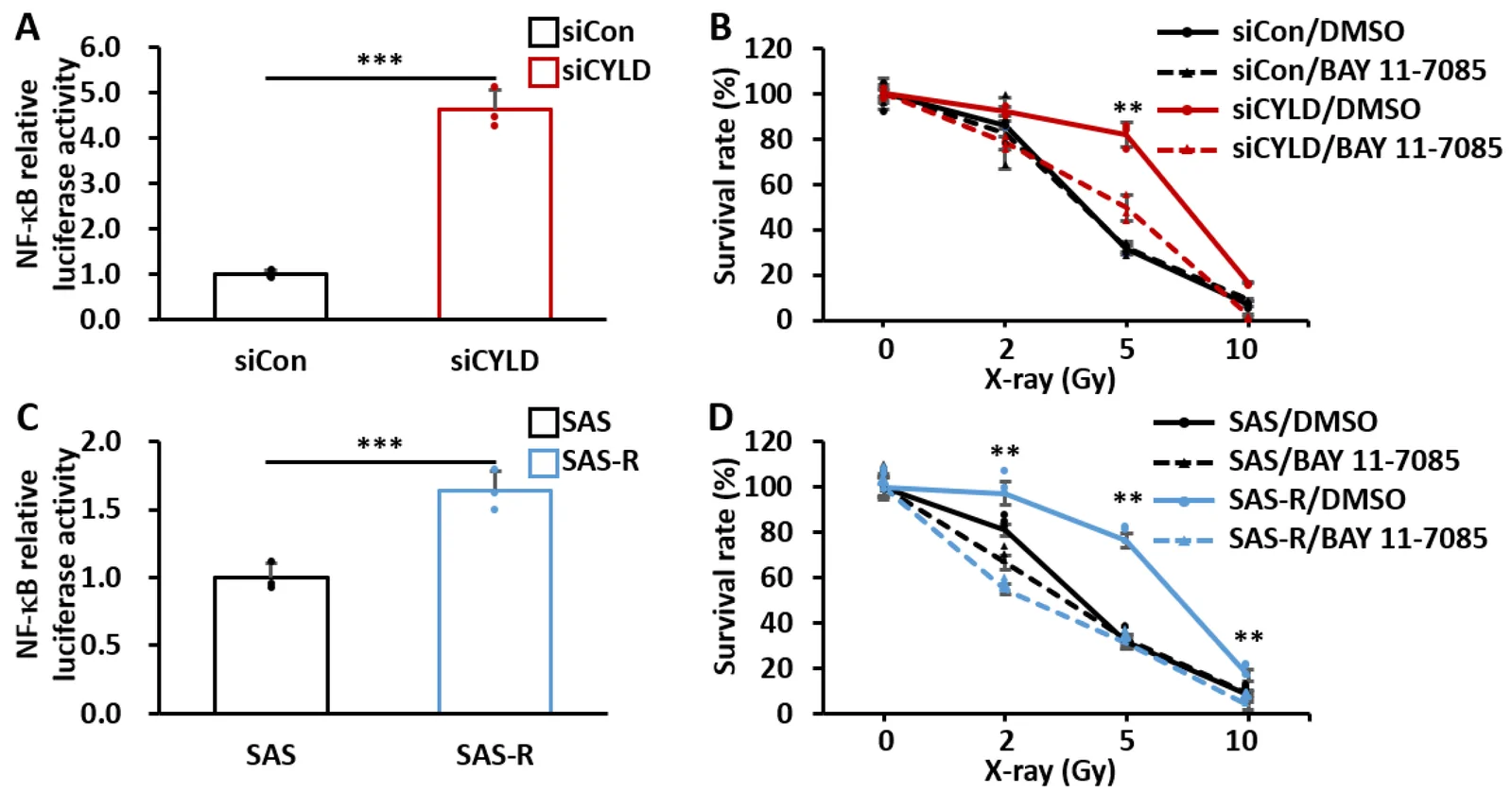
** Molecular mechanisms underlying the cross-resistance caused by low CYLD expression.** (A) NF-κB activity was assessed by using luciferase reporter assay in SAS cells transfected with siCYLD. (B) Cells were treated with BAY 11-7085 1 h before irradiation, and the survival rates were evaluated by using HDS assay in SAS cells transfected with siCYLD. (C) NF-κB activity was assessed by using luciferase reporter assay in SAS-R cells. (D) Cells were treated with BAY 11-7085 1 h before irradiation, and the survival rates were evaluated by using HDS assay in CRR cells. Values are means ± S.D. of triplicate samples. **p < 0.01, ***p<0.005 vs DMSO treated SAS-R or siCYLD in Tukey-Kramer method. Data are representative of 2-3 independent experiments.

**Figure 5:**
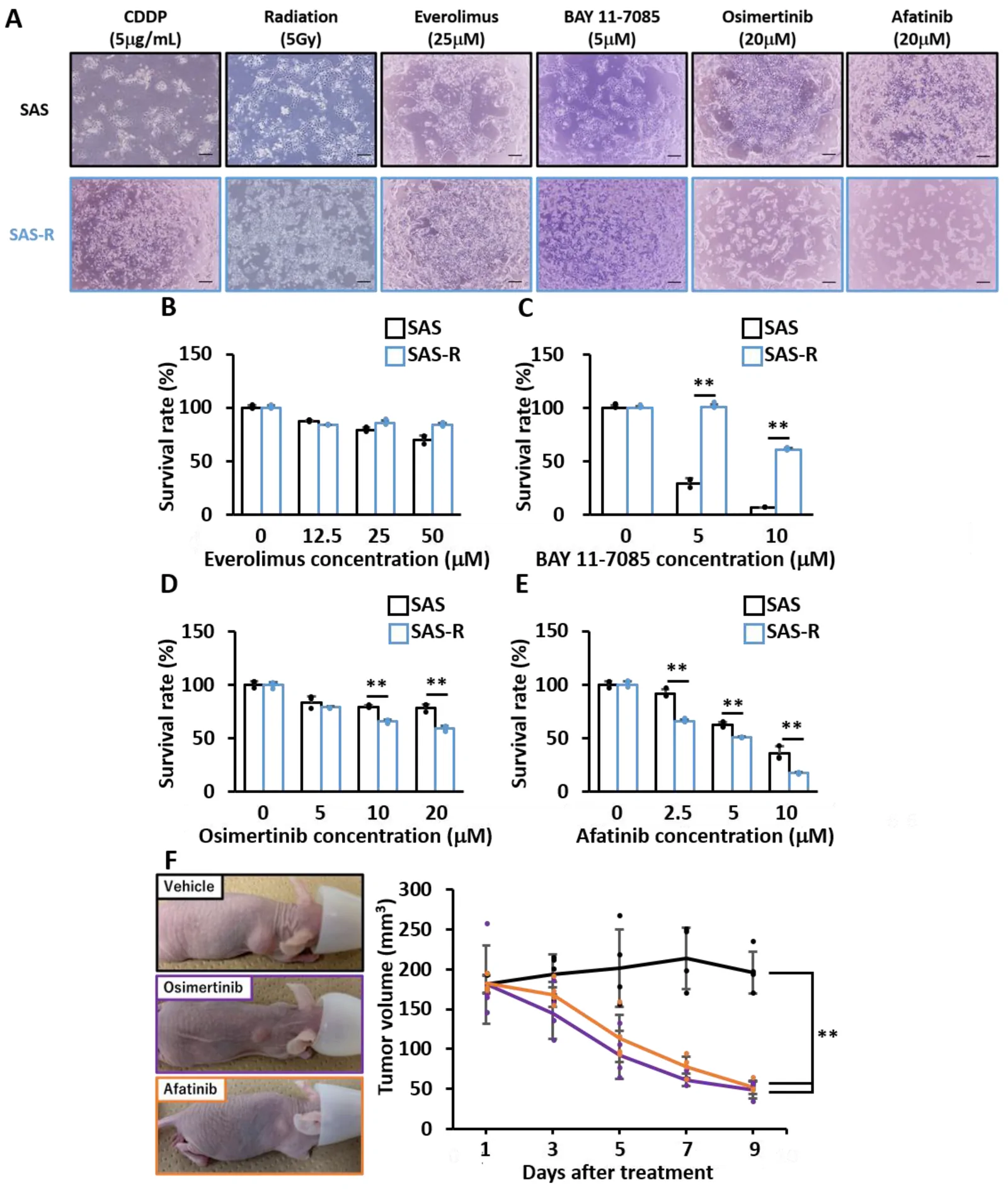
** EGFR-tyrosine kinase inhibitors are highly effective for OSCC with cross-resistance.** (A) Image of OSCC cells after irradiation and treatment with anti-cancer drugs such as CDDP, Everolimus, BAY 11-7085, osimertinib and afatinib. Scale bar shows 200 μm. (B-E) The survival rate of SAS-R 48h after Everolimus (0-50 μM), BAY 11-7085 (0-10μM), osimertinib (0-20 μM), Afatinib (0-20 μM) treatment compared with SAS. Values are means ± S.D. of triplicate samples. **p < 0.01 vs SAS in Tukey-Kramer method. (F) Image of mice at day 9 after reagents treatment. The tumor growth curves were measured after treatment. **p < 0.01 vs vehicle group in Tukey-Kramer method. Data are representative of 2-3 independent experiments.

**Figure 6:**
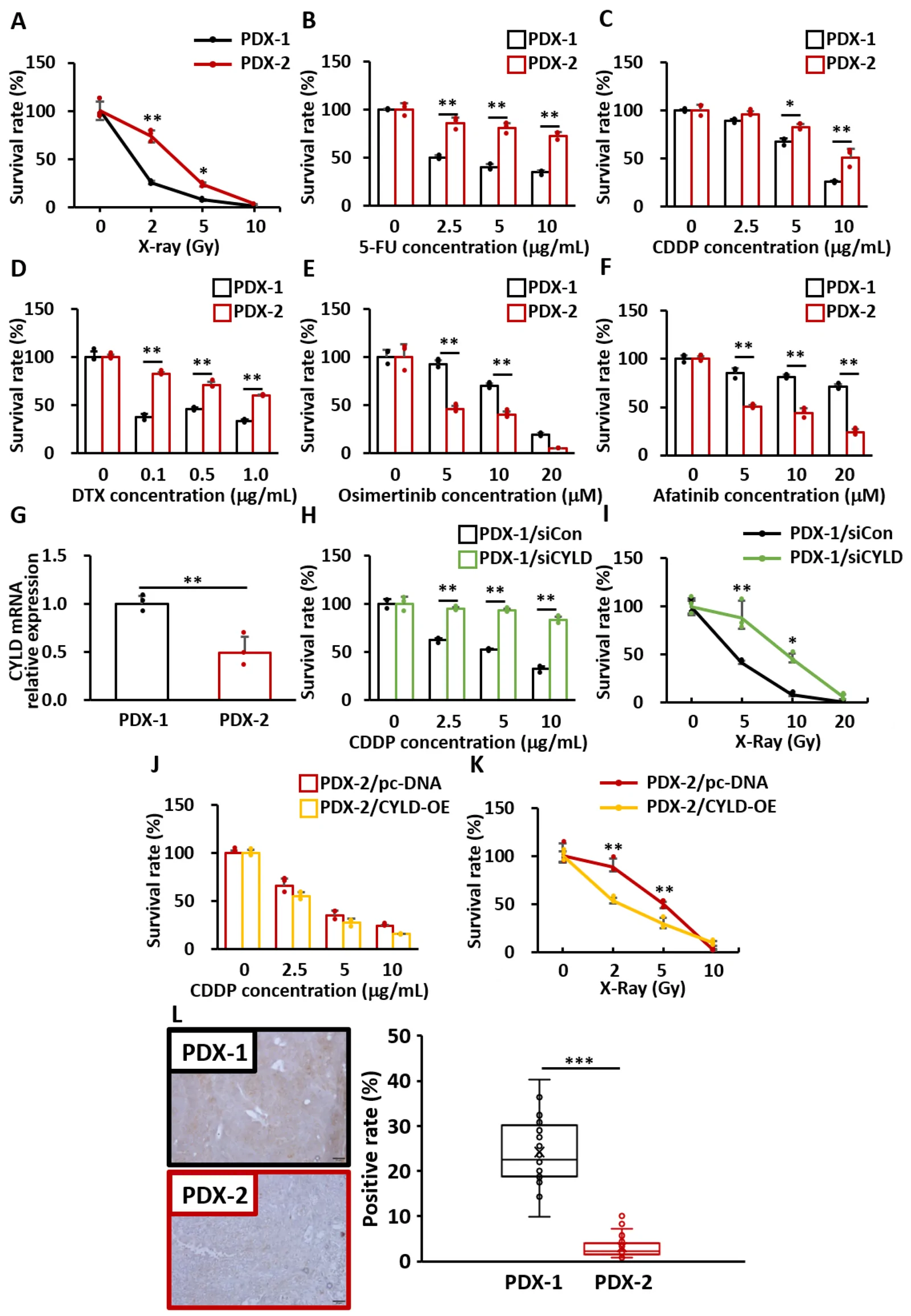
** Efficacy of EGFR-TKIs for patient-derived xenograft (PDX) models with cross-resistance by low CYLD expression.** (A) The cell survival rates were assessed after irradiation (0-10 Gy) by HDS assays in PDX-derived cells. (D-F) The survival rate 48h after treatment with CDDP (0-5µg/mL), 5-FU (0-5µg/mL), DTX (0-1µg/mL), osimertinib (0-20 μM), Afatinib (0-20 μM) by using cell counting kit-8. (G) Relative CYLD mRNA expression was assessed by RT-qPCR in PDX-derived cells. (H-I) The survival rates were assessed after irradiation and treatment of CDDP in CYLD knockdown PDX-1 cells. (J-K) The survival rates were assessed after irradiation and treatment of CDDP in CYLD overexpression PDX-2 cells. Values are means ± S.D. of triplicate samples. *p < 0.05, **p < 0.01 in Tukey-Kramer method. (L) The CYLD expression was assessed by immunohistochemical analysis in PDX tumor tissues. CYLD positive rates were calculated by Image J. Scale bar shows 50 µm. **p<0.01, ***p < 0.005 in Student's t-test. Data are representative of 2-3 independent experiments.

## Data Availability

The datasets used and/or analyzed during the current study are available from the corresponding author on reasonable request.

## Abbreviations

OSCC: Oral Squamous Cell Carcinoma
HNSCC: Head and Neck Squamous Cell Carcinoma
5-FU: Fluorouracil
CRR: Clinically Relevant Radio-resistant
CYLD: Cylindromatosis
USP: Ubiquitin Specific Protease
HDS: High-density survival
CDDP: cis-diamminedichloro-platinum (II)
DTX: Docetaxel
PTX: Paclitaxel
CDX: Cell line-Derived Xenograft
CPTAC: Clinical Proteomic Tumor Analysis Consortium
TCGA: The Cancer Genome Atlas
DSB: Double-strand break
HRR: Homologous Recombination Repair
EGFR: Epidermal Growth Factor Receptor
TKIs: tyrosine kinase inhibitors
PDX: Patient-Derived Xenograft
TRAF: Tumor Necrosis Factor
NEMO: NF-κB Essential Modulator
JNK: c-Jun N-terminal Kinase

## References

[1] Liu YP, Zheng CC, Huang YN (2021). Molecular mechanisms of chemo- and radiotherapy resistance and the potential implications for cancer treatment. MedComm.

[2] Esteller M (2008). Epigenetics in cancer. N Engl J Med.

[3] Sharma S, Kelly TK, Jones PA (2010). Epigenetics in cancer. Carcinogenesis.

[4] Goetz C, Raschka J, Wolff KD (2020). Hospital Based Quality of Life in Oral Cancer Surgery. Cancers.

[5] Pignon JP, Bourhis J, Domenge C (2000). Chemotherapy added to locoregional treatment for head and neck squamous-cell carcinoma: three meta-analyses of updated individual data. MACH-NC Collaborative Group. Meta-Analysis of Chemotherapy on Head and Neck Cancer. Lancet.

[6] Adelstein DJ, Saxton JP, Rybicki LA (2006). Multiagent concurrent chemoradiotherapy for locoregionally advanced squamous cell head and neck cancer: mature results from a single institution. J Clin Oncol.

[7] Vermorken JB, Mesia R, Rivera F (2008). Platinum-based chemotherapy plus cetuximab in head and neck cancer. NEJM.

[8] Colevas AD (2006). Chemotherapy options for patients with metastatic or recurrent squamous cell carcinoma of the head and neck. J Clin Oncol.

[9] Bonner JA, Harari PM, Giralt J (2006). Radiotherapy plus cetuximab for squamous-cell carcinoma of the head and neck. N Engl J Med.

[10] Bussink J, van der Kogel AJ, Kaanders JH (2008). Activation of the PI3-K/AKT pathway and implications for radioresistance mechanisms in head and neck cancer. Lancet Oncol.

[11] Han YK, Park HY, Park SG (2022). Promoter methylation of cancer stem cell surface markers as an epigenetic biomarker for prognosis of oral squamous cell carcinoma. Int J Mol Sci.

[12] Skvortsova I, Debbage P, Kumar V (2015). Radiation resistance: cancer stem cells (CSCs) and their enigmatic pro-survival signaling. Semin Cancer Biol.

[13] Tan Y, Wang Z, Xu M Oral squamous cell carcinomas: state of the field and emerging directions. 2023; 15: 44.

[14] Aebersold R, Mann M (2003). Mass spectrometry-based proteomics. Nature.

[15] Guo T, Steen JA, Mann M Mass-spectrometry-based proteomics: from single cells to clinical applications. 2025; 638: 901-911.

[16] Kuwahara Y, Mori M, Oikawa T (2010). The modified high-density survival assay is the useful tool to predict the effectiveness of fractionated radiation exposure. J Radiat Res.

[17] Massoumi R, Kuphal S, Hellerbrand C, et al Down-regulation of CYLD expression by Snail promotes tumor progression in malignant melanoma J Exp Med. 2009; 206: 221-232.

[18] Yuan Y, Liu L, Wang Y (2020). Reduced expression of CYLD promotes cell survival and inflammation in gefitinib-treated NSCLC PC-9 cells: Targeting CYLD may be beneficial for acquired resistance to gefitinib therapy. Cell Biol Int.

[19] Hayashi M, Jono H, Shinriki S (2014). Clinical significance of CYLD downregulation in breast cancer. Breast Cancer Res Treat.

[20] Guo J, Shinriki S, Su Y (2014). Hypoxia suppresses cylindromatosis (CYLD) expression to promote inflammation in glioblastoma possible link to acquired resistance to anti-VEGF therapy. Oncotarget.

[21] Kinoshita H, Okabe H, Beppu T (2013). CYLD downregulation is correlated with tumor development in patients with hepatocellular carcinoma. Mol Clin Oncol.

[22] Bignell GR, Warren W, Seal S (2000). Identification of the familial cylindromatosis tumour-suppressor gene. Nat Genet.

[23] Takahashi M, Rapley E, Biggs PJ Linkage and LOH studies in 19 cylindromatosis families show no evidence of genetic heterogeneity and refine the CYLD locus on chromosome 16q12-q13. Hum Genet. 200; 106: 58-65.

[24] Sacco JJ, Coulson JM, Clague MJ (2010). Emerging Roles of Deubiquitinases in Cancer-Associated Pathways. IUBMB Life.

[25] Lork M, Verhelst K, Beyaert R (2017). CYLD, A20 and OTULIN deubiquitinases in NF-kappa B signaling and cell death: so similar, yet so different. Cell Death Differ.

[26] Shinriki S, Jono H, Maeshiro M (2018). Loss of CYLD promotes cell invasion via ALK5 stabilization in oral squamous cell carcinoma. J Pathol.

[27] Masuda T, Saito N, Tomita M (2009). Unbiased quantitation of Escherichia coli membrane proteome using phase transfer surfactants. Mol Cell Proteomics.

[28] Masuda T, Tomita M, Ishihama Y (2008). Phase transfer surfactant-aided trypsin digestion for membrane proteome analysis. J Proteome Res.

[29] Rappsilber J, Mann M, Ishihama Y (2007). Protocol for micro-purification, enrichment, pre-fractionation and storage of peptides for proteomics using StageTips. Nat Protoc.

[30] Rappsilber J, Ishihama Y, Mann M (2003). Stop and go extraction tips for matrix-assisted laser desorption/ionization, nanoelectrospray, and LC/MS sample pretreatment in proteomics. Anal Chem.

[31] Gillet LC, Navarro P, Tate S (2012). Targeted data extraction of the MS/MS spectra generated by data-independent acquisition: a new concept for consistent and accurate proteome analysis. Mol Cell Proteomics.

[32] Okada S, Vaeteewoottacharn K, Kariya R (2019). Application of highly immunocompromised mice for the establishment of patient-derived xenograft (PDX) models. Cells.

[33] Vaeteewoottacharn K, Pairojkul C, Kariya R (2019). Establishment of highly transplantable cholangiocarcinoma cell lines from a patient-derived xenograft mouse model. Cells.

[34] Trompouki E, Hatzivassiliou E, Tsichritzis T (2003). CYLD is a deubiquitinating enzyme that negatively regulates NF-κB activation by TNFR family members. Nature.

[35] Brummelkamp TR, Nijman SMB, Dirac AMG (2003). Loss of the cylindromatosis tumour suppressor inhibits apoptosis by activating NF-κB. Nature.

[36] Kovalenko A, Chable-Bessia C, Cantarella G (2003). The tumour suppressor CYLD negatively regulates NF-κB signalling by deubiquitination. Nature.

[37] Tauriello DVF, Haegebarth A, Kuper I (2010). Loss of the Tumor Suppressor CYLD Enhances Wnt/β-Catenin Signaling through K63-Linked Ubiquitination of Dvl. Mol. Cell.

[38] Reiley W, Zhang M, Sun SC (2004). Negative Regulation of JNK Signaling by the tumor suppressor CYLD. J Biol Chem.

[39] Tesio M, Tang Y, Müdder K (2015). Hematopoietic stem cell quiescence and function are controlled by the CYLD-TRAF2-p38MAPKpathway. J Exp Med.

[40] Chen Y, Wang Z, Wang P (2014). CYLD negatively regulates Hippo signaling by limiting Hpo phosphorylation in Drosophila. Biochem Biophys Res Commun.

[41] Rajan N, Elliott RJR, Smith A (2014). The cylindromatosis gene product, CYLD, interacts with MIB2 to regulate notch signalling. Oncotarget.

[42] Almeida LO, Abrahao AC, Rosselli-Murai LK (2014). NFkappaB mediates cisplatin resistance through histone modifications in head and neck squamous cell carcinoma (HNSCC). FEBS Open Bio.

[43] Liu R, Shinriki S, Maeshiro M (2022). The Tumour Suppressor CYLD Is Required for Clathrin-Mediated Endocytosis of EGFR and Cetuximab-Induced Apoptosis in Head and Neck Squamous Cell Carcinoma. Cancers.

[44] Ayumi K, Shinriki S, Kai M (2022). Potential use of EGFR-targeted molecular therapies for tumor suppressor CYLD-negative and poor prognosis oral squamous cell carcinoma with chemoresistance. Cancer Cell Int.

[45] Wang Z, Wang Q, Xu G (2020). The long noncoding RNA CRAL reverses cisplatin resistance via the miR-505/CYLD/AKT axis in human gastric cancer cells. RNA Biol.

[46] Qiu C, Zhang S, Gao S (2020). Bisdemethoxycurcumin Inhibits Hepatocellular Carcinoma Proliferation Through Akt Inactivation via CYLD-Mediated Deubiquitination. Drug Des Devel Ther.

[47] Yuichiro M, Ryoji Y, Kenta K (2022). The antioxidative stress regulator Nrf2 potentiates radioresistance of oral squamous cell carcinoma accompanied with metabolic modulation. Springer nature.

[48] Kuwahara Y, Roudkenar MH, Suzuki M (2016). The Involvement of Mitochondrial Membrane Potential in Cross-Resistance Between Radiation and Docetaxel. Int J Radiat Oncol Biol Phys.

[49] Alsahafi E, Begg K, Amelio I (2019). Clinical update on head and neck cancer: molecular biology and ongoing challenges. Cell Death Dis.

[50] Laimer K, Spizzo G, Gastl G (2007). High EGFR expression predicts poor prognosis in patients with squamous cell carcinoma of the oral cavity and oropharynx: a TMA-based immunohistochemical analysis. Oral Oncol.

[51] Solomon B, Young RJ, Rischin D (2018). Head and neck squamous cell carcinoma: Genomics and emerging biomarkers for immunomodulatory cancer treatments. Semin Cancer Biol.

[52] Chen BPC, Uematsu N, Kobayashi J (2007). Ataxia telangiectasia mutated (ATM) is essential for DNA-PKcs phosphorylations at the Thr-2609 cluster upon DNA double strand break. J Biol Chem.

[53] Dittmann K, Mayer C, Fehrenbacher B (2005). Radiation-induced epidermal growth factor receptor nuclear import is linked to activation of DNA-dependent protein kinase. J Biol Chem.

[54] Cochicho D, Esteves S, Rito M (2022). PIK3CA Gene Mutations in HNSCC: Systematic Review and Correlations with HPV Status and Patient Survival. Cancers.

[55] Kang H, Kiess A, Chung CH (2015). Emerging biomarkers in head and neck cancer in the era of genomics. Nat Rev Clin Oncol.

[56] Ito K, Ota A, Ono T (2016). Inhibition of Nox1 induces apoptosis by attenuating the AKT signaling pathway in oral squamous cell carcinoma cell lines. Oncol Rep.

[57] Zhang X, Sun K, Gan R (2024). WNT3 promotes chemoresistance to 5-Fluorouracil in oral squamous cell carcinoma via activating the canonical β-catenin pathway. BMC Cancer.

[58] Li L, Liu H, Wang C (2016). Overexpression of β-Catenin Induces Cisplatin Resistance in Oral Squamous Cell Carcinoma. Biomed Res Int.

[59] Huang J, Li H, Yang Z (2024). SALL4 promotes cancer stem-like cell phenotype and radioresistance in oral squamous cell carcinomas via methyltransferase-like 3-mediated m6A modification. Cell Death Dis.

[60] Zhu R, Mao Y, Xu X (2024). HOXA1 silencing inhibits cisplatin resistance of oral squamous cell carcinoma cells via IκB/NF-κB signaling pathway. Anticancer Drugs.

[61] Chiu HW, Lee HL, Lee HH (2024). AIM2 promotes irradiation resistance, migration ability and PD-L1 expression through STAT1/NF-κB activation in oral squamous cell carcinoma. J Transl Med.

[62] Chiu HW, Lin CH, Lu HW (2024). Guanylate binding protein 5 triggers NF-κB activation to foster radioresistance, metastatic progression and PD-L1 expression in oral squamous cell carcinoma. Clin Immunol.

[63] Lin Y, Wang L, Luo W (2020). CYLD Promotes Apoptosis of Nasopharyngeal Carcinoma Cells by Regulating NDRG1. Cancer Manag Res.

[64] Naoki S, Kuramitsu M, Komure K (2019). Loss of tumor Figressor CYLD expression triggers cisplatin resistance in oral squamous cell carcinoma. Int J Mol Sci.

[65] Wang CY, Guttridge DC, Mayo MW (1999). NF-κB Induces Expression of the Bcl-2 Homologue A1/Bfl-1 To Preferentially Suppress Chemotherapy-Induced Apoptosis. Mol Cell Biol.

[66] Khoshnan A, Tindell C, Laux I (2000). The NF-kappa B cascade is important in Bcl-xL expression and for the anti-apoptotic effects of the CD28 receptor in primary human CD4+ lymphocytes. J Immunol.

[67] Catz SD, Johnson JL (2001). Transcriptional regulation of bcl-2 by nuclear factor kappa B and its significance in prostate cancer. Oncogene.

[68] Budke B, Zhong A, Sullivan K (2022). Noncanonical NF-κB factor p100/p52 regulates homologous recombination and modulates sensitivity to DNA-damaging therapy. Nucleic Acids Res.

[69] Volcic M, Karl S, Baumann B (2012). NF-κB regulates DNA double-strand break repair in conjunction with BRCA1-CtIP complexes. Nucleic Acids Res.

