# Supplementary material for: CYLD: A Key Factor and Novel Predictive Biomarker of Cross-Resistance to Chemotherapy and Radiotherapy in Oral Squamous Cell Carcinoma
Source: Int J Biol Sci. 2026 Oct 2;22(15):8653–67. doi: 10.7150/ijbs.138201 (PMC13686046; doi:10.7150/ijbs.138201)
Supplement: Supplementary file 1 [file ijbsv22p8653s1.pdf]

Supplementary Figure 1

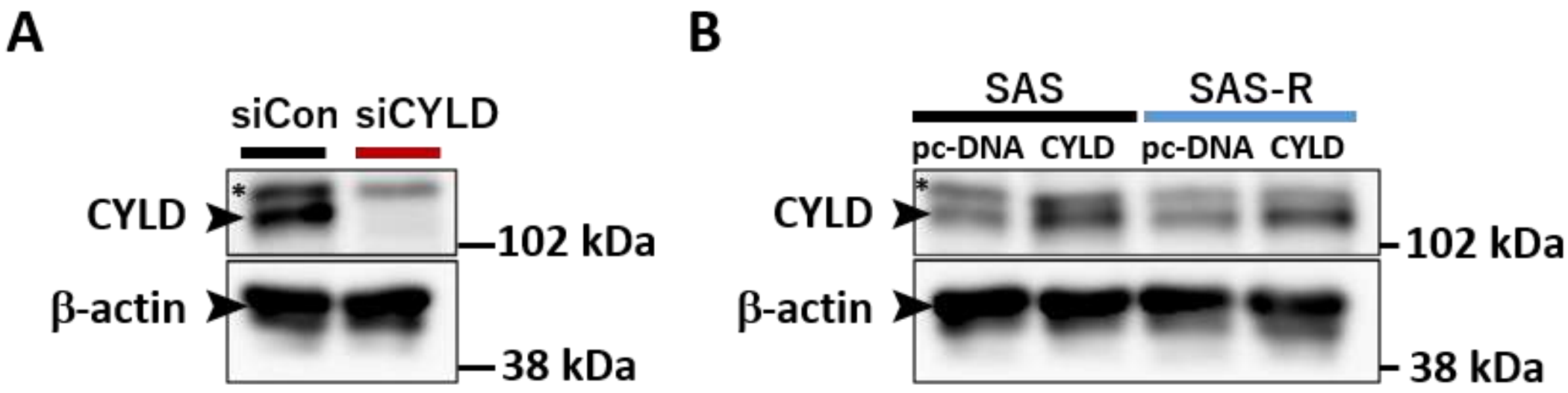

Supplementary Figure 1. Validation of CYLD knockdown and overexpression by western blotting.

(A) CYLD protein expression was examined by western blotting following transfection of CYLD siRNA into SAS cells. (B) CYLD protein expression was examined by western blotting following transfection of CYLD expression plasmid into SAS and SAS-R cells. The asterisk (*) indicates a non-specific band. Data are representative of 3 independent experiments.

Supplementary Figure 2

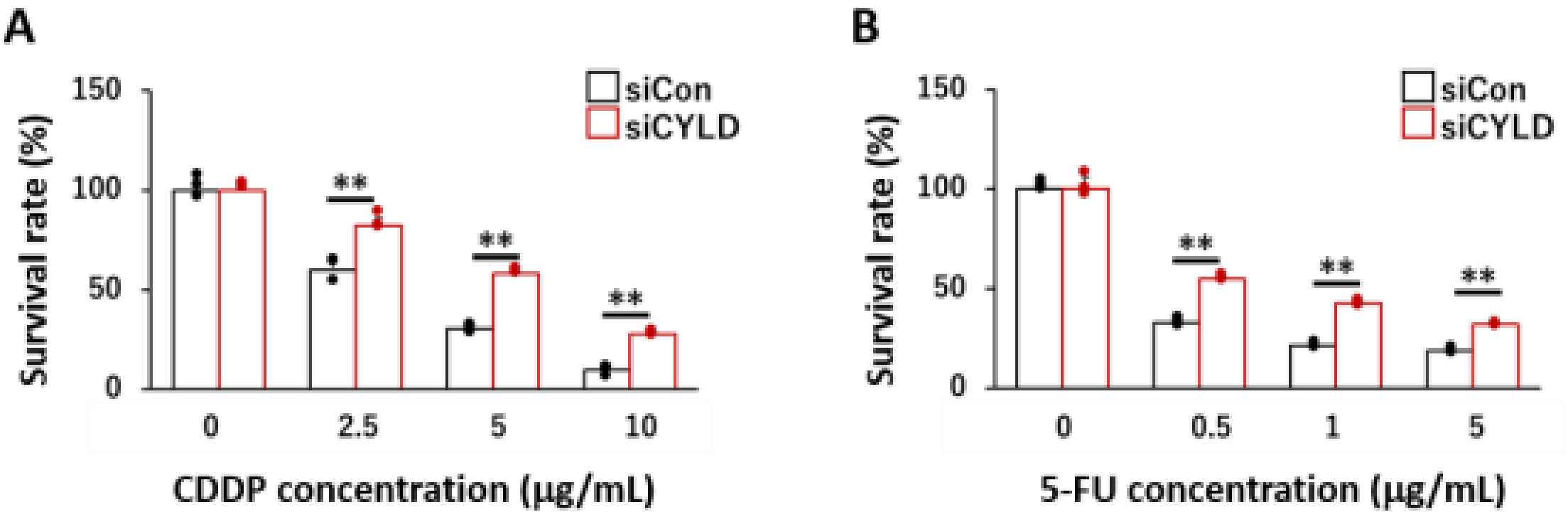

Supplementary Figure 2. CYLD downregulation induced cytotoxic drugs resistance such as CDDP and 5-FU.

SAS cells were transfected with control siRNA (siCon) or CYLD-specific siRNA (siCYLD). (A-B) The survival rates of siCYLD were evaluated after treatment of CDDP (0-10 μg/mL)) and 5-FU (0-5 μg/mL)) by using HDS assay. Values are means ± S.D. of triplicate samples. *p<0.05, **p < 0.01 vs siCon in Tukey–Kramer method. These experiments were independently repeated three times with similar results.
